# Efficient decoding algorithms for generalized hidden Markov model gene finders

**DOI:** 10.1186/1471-2105-6-16

**Published:** 2005-01-24

**Authors:** William H Majoros, Mihaela Pertea, Arthur L Delcher, Steven L Salzberg

**Affiliations:** 1 Bioinformatics Department, The Institute for Genomic Research, 9712 Medical Center Drive, Rockville, MD, USA

## Abstract

**Background:**

The Generalized Hidden Markov Model (GHMM) has proven a useful framework for the task of computational gene prediction in eukaryotic genomes, due to its flexibility and probabilistic underpinnings. As the focus of the gene finding community shifts toward the use of homology information to improve prediction accuracy, extensions to the basic GHMM model are being explored as possible ways to integrate this homology information into the prediction process. Particularly prominent among these extensions are those techniques which call for the simultaneous prediction of genes in two or more genomes at once, thereby increasing significantly the computational cost of prediction and highlighting the importance of speed and memory efficiency in the implementation of the underlying GHMM algorithms. Unfortunately, the task of implementing an efficient GHMM-based gene finder is already a nontrivial one, and it can be expected that this task will only grow more onerous as our models increase in complexity.

**Results:**

As a first step toward addressing the implementation challenges of these next-generation systems, we describe in detail two software architectures for GHMM-based gene finders, one comprising the common array-based approach, and the other a highly optimized algorithm which requires significantly less memory while achieving virtually identical speed. We then show how both of these architectures can be accelerated by a factor of two by optimizing their content sensors. We finish with a brief illustration of the impact these optimizations have had on the feasibility of our new homology-based gene finder, TWAIN.

**Conclusions:**

In describing a number of optimizations for GHMM-based gene finders and making available two complete open-source software systems embodying these methods, it is our hope that others will be more enabled to explore promising extensions to the GHMM framework, thereby improving the state-of-the-art in gene prediction techniques.

## Background

Generalized Hidden Markov Models have seen wide use in recent years in the field of computational gene prediction. A number of *ab initio *gene-finding programs are now available which utilize this mathematical framework internally for the modeling and evaluation of gene structure [1-6], and newer systems are now emerging which expand this framework by simultaneously modeling two genomes at once, in order to harness the mutually informative signals present in homologous gene structures from recently diverged species. As greater numbers of such genomes become available, it is tempting to consider the possibility of integrating all this information into increasingly complex models of gene structure and evolution.

Notwithstanding our eagerness to utilize this expected flood of genomic data, methods have yet to be demonstrated which can perform such large-scale parallel analyses without requiring inordinate computational resources. In the case of Generalized Pair HMMs (GPHMMs), for example, the only systems in existence of which we are familiar make a number of relatively restrictive assumptions in order to reduce the computational complexity of the problem to a more tolerable level [7,8,15]. Yet, even these systems are currently capable of handling no more than two genomes at once. If larger numbers of genomes are to be simultaneously integrated into the gene prediction process in a truly useful manner, then it is reasonable to suggest that new methods will be needed for efficient modeling of parallel gene structures and their evolution. Assuming for now that these methods are likely to continue to build on the basic GHMM framework, we feel it is important that efficient methods of GHMM implementation be properly disseminated for the benefit of those who are to work on this next generation of eukaryotic gene finders.

### Modeling genes with a GHMM

A Hidden Markov Model (HMM) is a state-based generative model which transitions stochastically from state to state, emitting a single symbol from each state. A GHMM (or *semi-Markov *model) generalizes this scenario by allowing individual states to emit strings of symbols rather than one symbol at a time [9,10]. A GHMM is parameterized by its transition probabilities, its state duration (i.e., feature length) probabilities, and its state emission probabilities. These probabilities influence the behavior of the model in terms of which sequences are most likely to be emitted and which series of states are most likely to be visited by the model as it generates its output.

Eukaryotic gene prediction entails the parsing of a DNA sequence into a set of putative CDSs (*coding segments*, hereafter referred to informally as "genes") and their corresponding exon-intron structures [11]. Thus, the problem of eukaryotic gene prediction can be approximately stated as one of parsing sequences over the nucleotide alphabet Σ = {A,C,G,T} according to the regular expression:

Σ*(ATGΣ*(GTΣ*AG)*Σ*Γ)*Σ*,     (1)

where the *signals *(start and stop codons, donors, and acceptors) have been underlined for clarity, and where Γ = {TAG,TGA,TAA} represents a stop codon. (The actual nucleotides comprising these signals may differ between organisms; we have given the most common ones). An additional constraint not explicitly represented in Formula 1 is that the number of non-intron nucleotides between the start and stop codons of a single gene must be a multiple of three, and furthermore, if these nucleotides are aggregated into a discrete number of nonoverlapping triples, or *codons*, then none of these codons must be a stop codon, other than the stop codon which terminates the gene. Note that the Σ* terms in Formula 1 permit the occurrence of *pseudo-signals *– e.g., an ATG triple which does not comprise a true start codon. Gene prediction with a GHMM thus entails parsing with an ambiguous stochastic regular grammar; the challenge is to find the most probable parse of an input sequence, given the GHMM parameters and the input sequence.

In the case of simple Hidden Markov Models, this optimal parsing (or *decoding*) problem can be solved with the well-known *Viterbi algorithm*, a dynamic programming algorithm with run time linear in the sequence length (for a fixed number of states) [12]. A modified Viterbi algorithm is required in the case of GHMMs, since each state can now emit more than one symbol at a time [2], resulting in the following optimization problem:


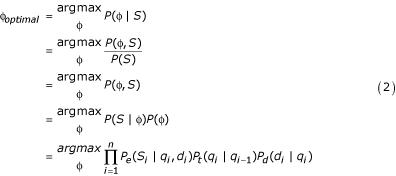


where φ is a *parse *of the sequence consisting of a series of states *q*_*i *_and state durations *d*_*i*_, 0≤*i*≤*n*, with each state *q*_*i *_emitting subsequence *S*_*i *_of length *d*_*i*_, so that the concatenation of all *S*_0_*S*_1_...*S*_*n *_produces the complete output sequence *S *(but note that states *q*_0 _and *q*_*n *_are *silent*, producing no output). *P*_*e*_(*S*_*i*_|*q*_*i*_,*d*_*i*_) denotes the probability that state *q*_*i *_emits subsequence *S*_*i*_, given duration *d*_*i*_; *P*_*t*_(*q*_*i*_|*q*_*i*-1_) is the probability that the GHMM transitions from state *q*_*i*-1 _to state *q*_*i*_; and *P*_*d*_(*d*_*i*_|*q*_*i*_) is the probability that state *q*_*i *_has duration *d*_*i*_. The *argmax *is over all parses of the DNA sequence into well-formed exon-intron structures; hence, the problem is one of finding the parse which maximizes the product in Equation 2.

## Implementation

### The PSA decoding algorithm

The approach commonly used in GHMM gene finders for evaluating Equation 2 is to allocate several arrays, one per variable-length feature state, and to evaluate the arrays left-to-right along the length of the input sequence according to a dynamic programming algorithm, which we will detail below. We refer to this approach as the *Prefix Sum Arrays *(PSA) approach, since the values in the aforementioned arrays represent cumulative scores for prefixes of the sequence.

**Figure 1:**
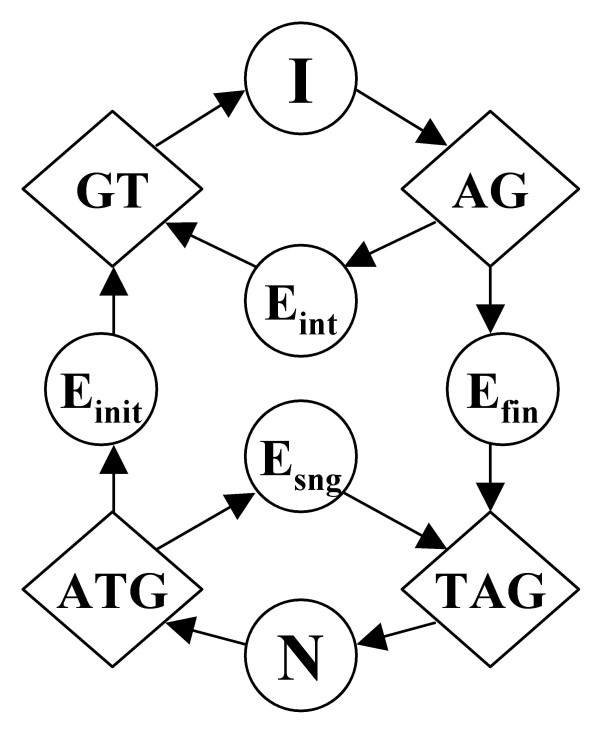
**An example GHMM topology. **Diamonds represent signal states (for fixed-length features) and circles represent content states (for variable-length features). Allowable transitions are shown with arrows. ATG = start codon, TAG = stop codon, GT = donor splice site, AG = acceptor splice site, N = intergenic region, I = intron, E_init _= initial exon, E_int _= internal exon, E_fin _= final exon, E_sng _= single exon gene. The denoted machine operates by transitioning stochastically from state to state, emitting a gene feature of a particular type upon entering a given state.

Without loss of generality, let us consider the GHMM structure depicted in Figure 1. Although individual GHMMs will differ from this particular structure on specific points, the model in Figure 1 is general enough to serve as a concrete example as we illustrate the operation of the algorithm.

The diamonds denote the states for fixed length features (ATG = start codon, TAG = stop codon, GT = donor, AG = acceptor) and the circles denote states for variable length features (N = intergenic, I = intron, E_sng _= single exon, E_init _= initial exon, E_int _= internal exon, E_fin _= final exon). This model generates genes only on the forward strand of the DNA; to obtain a double-stranded model one can simply mirror the structure and link the forward and reverse models through a single merged intergenic state.

Associated with each diamond state is a *signal sensor *such as a *weight matrix *(WMM) or some other fixed-length model (e.g., a WAM, WWAM, MDD tree, etc.) [13], and with each circular state is associated a variable-length *content sensor*, such as a *Markov chain *(MC) or an *Interpolated Markov Model *(IMM) [14].

For the purposes of illustration, we will consider only the simplest of each model type, since the more complex model types commonly in use can in general be handled generically within the GHMM framework. The simplest fixed-length model is the WMM:


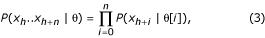


where *x*_*h*_..*x*_*h*+*n *_denotes the subsequence currently within a sliding (*n *+ 1)-element window, called the *context window*, and *P*(*x*|θ[*i*]) denotes the probability of nucleotide *x *occurring at position *i *within the window, for model θ. In practice, all of the probabilities described in all of these models are represented in log space (to reduce the incidence of numerical underflow on the computer), so that products of probabilities can be replaced with sums of their logs.

The simplest variable-length model used in practice is the Markov chain. An *n*^th^-order Markov chain *M *for state *q*_*i *_would evaluate the probability *P*(*S*_*i*_|*q*_*i*_,*d*_*i*_) of a putative feature *S*_*i *_according to:


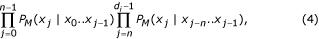


where *x*_*j *_is the *j*^th ^nucleotide in the sequence of the putative feature, *d*_*i *_is the length of that feature, and *P*_*M*_(*x*_*j*_|*x*_*j*-*n*_..*x*_*j*-1_) is the probability of nucleotide *x*_*j *_conditional on the identities of its *n *predecessor nucleotides, according to *content model M*. As with the fixed-length model described above, this computation is typically done in log space.

**Figure 2:**
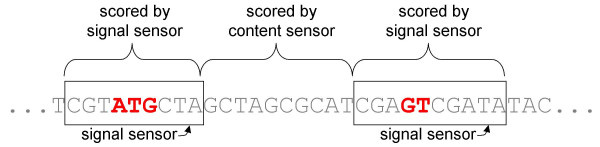
**Non-overlapping of content and signal sensors. **Fixed-length features such as start codons and donor sites are detected by signal sensors, which are used to score an entire context window surrounding the signal. To avoid double-counting, content sensors score only the nucleotides strictly between two signal sensors. In this example, the CTA at the end of the start codon sensor window and the CGA at the beginning of the donor site sensor window are not scored by the exon content sensor, even though they are part of the putative exon, since those bases are already scored by the signal sensors.

In scoring the signals and content regions of a putative gene parse, it will be important for us to carefully differentiate between the nucleotides which are scored by a signal sensor and those which are scored by a content sensor in a putative parse. As shown in Figure 2, the content and signal regions must partition the sequence into non-overlapping segments; allowing overlaps would result in double-counting of nucleotide probabilities, which can lead to undesirable biases in the decoding algorithm.

**Figure 3:**
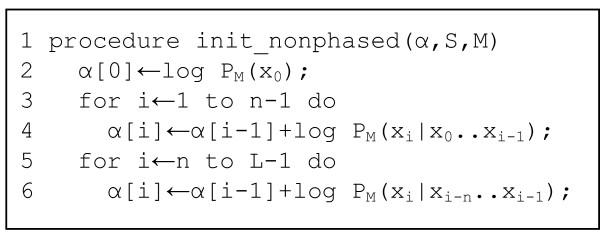
**The init_nonphased() algorithm. **Initialization of a noncoding array α, given a sequence *S *= *x*_0_..*x*_*L*-1 _and *n*^th^-order Markov chain *M*. Note that all parameters are assumed passed by reference. The procedure initializes each array element to the log probability of the nucleotide at the corresponding position in the sequence, conditional on some number of preceding bases.

The first step of the PSA algorithm is to compute a prefix sum array for each content sensor. For noncoding states (introns and intergenic) this can be formalized as shown in Figure 3.

**Figure 4:**
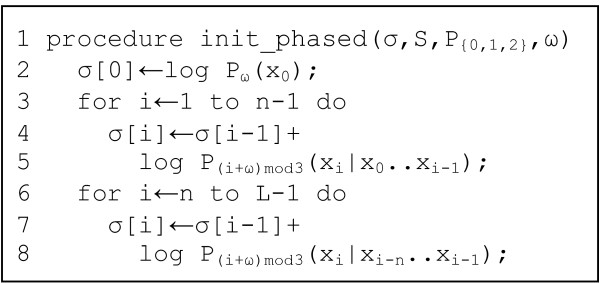
**The init_phased() algorithm. **Initialization of a single exon array σ, given a sequence *S *= *x*_0_..*x*_*L*-1_, a set of three Markov chains *P*_{0,1,2}_, and *initial phase *(i.e., phase of the first array element) ω. All parameters are assumed to be passed by reference. This procedure is similar to init_nonphased(), except that the conditional probabilities are computed in a phase-specific manner by the appropriate member of the three-periodic Markov chain.

In the case of exon states, it is important to capture the different statistical properties present in the three codon positions, referred to as *phase 0*, *phase 1*, and *phase 2*. We employ three Markov chains, *M*_0_, *M*_1_, and *M*_2_, corresponding to these three phases. Together, these three chains constitute a *three-periodic Markov chain*, *M*_{0,1,2}_. Exon states then require three arrays, each of which can be initialized using the procedure shown in Figure 4.

In this way, we can initialize the three arrays α_i, 0_, α_i, 1_, and α_i, 2 _for an exon state *q*_*i *_as follows:

**for **ω ← 0 **to **2 **do**

   init_phased(α_i,ω_, S, M_{0, 1, 2}_,ω) ;

The individual chains *M*_0_, *M*_1_, and *M*_2 _comprising *M*_{0,1,2} _are applied in periodic fashion within the procedure init_phased() to compute conditional probabilities of successive nucleotides along the length of the array. The three arrays are phase-shifted by one from each other, with each element in the array storing the cumulative score of the prefix up to the current nucleotide. The first nucleotide is taken to be in phase ω for array α_*i*_,ω. Initializing the arrays for reverse-strand states can be achieved by simply reverse-complementing the DNA sequence and then reversing the order of the resulting arrays (keeping in mind later that the reverse-strand arrays tabulate their sums from the right, rather than the left, and that ω is the phase of the last array entry rather than the first).

Once the prefix sum arrays have been initialized for all variable-duration states, we make another left-to-right pass over the input sequence to look for all possible matches to the fixed-length states, via the signal sensors. In general, a signal sensor θ models the statistical biases of nucleotides at fixed positions surrounding a signal of a given type, such as a start codon. Whenever an appropriate consensus is encountered (such as ATG for the start codon sensor), the signal sensor's fixed-length window is superimposed around the putative signal (i.e., with a margin of zero or more nucleotides on either side of the signal consensus) and evaluated to produce a logarithmic signal score *R*_*S *_= **log ***P*(*x*_*h*.._*x*_*h*+*n*-1_|θ), where *h *is the position of the beginning of the window and *n *is the window length. If signal *thresholding *is desired, *R*_*S *_can be compared to a pre-specified threshold and those locations scoring below the threshold can be eliminated from consideration as putative signals.

The remaining candidates for signals of each type are then inserted into a type-specific *signal queue *for consideration later as possible predecessors of subsequent signals in a putative gene model. As each new signal is encountered, the optimal predecessors for the signal are selected from among the current contents of the signal queues, using a scoring function described below. In the example (forward strand) GHMM depicted in Figure 1, the possible (predecessor→successor) patterns are:

ATG→TAG

ATG→GT

GT→AG

AG→GT

AG→TAG

TAG→ATG

Associated with each of these patterns is a transition probability, *P*_*t*_(*q*_*i*_|*q*_*i*-1_), which is included in the scoring of a possible predecessor; this probability can be accessed quickly by indexing into a two-dimensional array. The logarithmic transition score will be denoted *R*_*T*_(*q*_*i*-1_,*q*_*i*_) = **log ***P*_*t*_(*q*_*i*_|*q*_*i*-1_).

The distance from a prospective predecessor to the current signal is also included in the evaluation in the form of *P*_*d*_(*d*_*i*_|*q*_*i*_) for distance (=duration) *d*_*i *_and signal type (=state) *q*_*i*_. This probability can usually be obtained relatively quickly, depending on the representation of the duration distributions. If the distributions have been fitted to a curve with a simple algebraic formula, then evaluation of the formula is typically a constant-time operation. If a histogram is instead maintained, then a binary search is typically required to find the histogram interval containing the given distance. We denote the logarithmic duration score *R*_*D*_(*q*_*i*_,*q*_*j*_) = **log ***P*_*d*_(*d*_*i*_|*q*_*i*:*j*_) where *d*_*i *_is the length of the content region delimited by signals *q*_*i *_and *q*_*j*_, and *q*_*i*:*j *_is the variable-length state corresponding to that content region.

Following Equation 2, the final component of the scoring function is the emission probability *P*_*e*_(*S*_*i*_|*q*_*i*_,*d*_*i*_). For a fixed-length state, this is simply the score produced by the signal sensor. For a variable-length state *q*_*i*_, *P*_*e *_can be evaluated very quickly by indexing into the prefix sum array α_*i*,γ _for state *q*_*i *_and phase γ at the appropriate indices for the two signals and simply performing subtraction:

*R*_*C*_(*s*_pred_,*s*_cur_,ω) ← α_*i*,γ _[*wpos*(*s*_cur_) - 1] - α_*i*,γ _[*wpos*(*s*_pred_) + *wlen*(*s*_pred_) - 1],     (5)

where *wpos*(*s*) is the 0-based position (within the full input sequence) of the first nucleotide in the context window for signal *s*, *wlen*(*s*) is the length of the context window for signal *s*, and *s*_pred _and *s*_cur _are the predecessor and current signals, respectively. In the case of coding features, γ is the phase of the array and ω = (γ + *pos*(*s*_cur_))**mod**3 is the phase of *s*_cur_, for *pos*(*s*_cur_) the position of the leftmost consensus base of *s*_cur_. For reverse-strand features, since the prefix sum arrays tabulate their sums from the right instead of the left, the subtraction must be reversed:

*R*_*C*_(*s*_pred_,*s*_cur_,ω) ← α_*i*,γ_[*wpos*(*s*_pred_) + *wlen*(*s*_pred_)] - α_*i*,γ_[*wpos*(*s*_cur_)],     (6)

and ω = (γ +*L *- *pos*(*s*_cur_) - 1)**mod**3, for *L *the sequence length. For noncoding features, the phases can be ignored when computing *R*_*C*_, since there is only one array per noncoding state.

The resulting optimization function is:


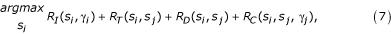


for current signal *s*_*j *_and predecessor signal *s*_*i*_; *R*_*I*_(*s*_*i*_,γ_*i*_) denotes the logarithmic inductive score for signal *s*_*i *_in phase γ_*i*_. For forward-strand coding features, the phases γ_*i *_and γ_*j *_are related by:

γ_*i *_= (γ_*j *_- Δ)**mod**3,     (8)

for Δ the putative exon length, or, equivalently,

γ_*j *_= (γ_*i *_+ Δ)**mod**3.     (9)

These relations can be converted to the reverse strand by swapping + and -. For introns, γ_*i *_= γ_*j*_. For intergenic features, the phase will always be 0 for a forward strand signal and 2 for a reverse strand signal (since on the reverse strand the leftmost base of a 3-base signal would be in phase 2).

The result of Equation 7 is the optimal predecessor for signal *s*_*j*_. This scoring function is evaluated for all appropriate predecessor signals, which are readily available in one or more queues, as mentioned above. A pointer called a *trellis link *is then created, pointing from the current signal to its optimal predecessor. In the case of those signals that can terminate an exon or an intron, three optimal predecessors must be retained, one for each phase. The inductive score *R*_*I*_(*s*_*j*_,γ_*j*_) of the new signal *s*_*j *_is then initialized from the selected predecessor *s*_*i *_as follows:

*R*_*I*_(*s*_*j*_, γ_*j*_) ← *R*_*I*_(*s*_*i*_, γ_*j*_) + *R*_*T*_(*s*_*i*_, *s*_*j*_) + *R*_*D*_(*s*_*i*_, *s*_*j*_) + *R*_*C*_(*s*_*i*_, *s*_*j*_, γ_*j*_) + *R*_*S*_(*s*_*j*_),     (10)

where *R*_*S*_(*s*_*j*_) is the logarithmic score produced by the signal sensor for signal *s*_*j*_.

**Figure 5:**
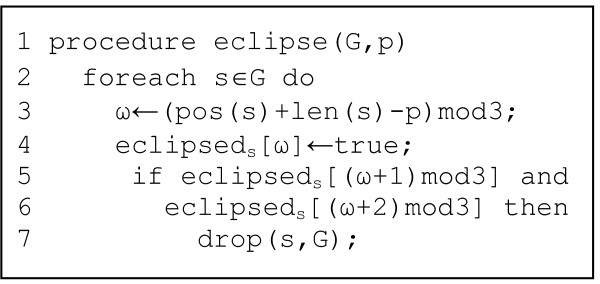
**The eclipse() algorithm. **Eclipsing signals in queue *G *when a stop codon has been encountered at position *p*. All parameters are assumed to be passed by reference. pos(s) is the position of the first base of the signal's consensus sequence (e.g., the A in ATG). len(s) is the length of the signal's consensus sequence (e.g., 3 for ATG). The procedure operates by computing the phase ω in which each signal is eclipsed by the stop codon, and then identifies those signals which are now eclipsed in all three phases. Any signal eclipsed in all three phases is then dropped from the queue, since any exon starting at that signal and extending up to the current position in the sequence would have an in-frame stop codon.

A final step to be performed at each position along the input sequence is to drop from each queue any signal that has been rendered unreachable from all subsequent positions due to intervening stop codons. Except for the final stop codon of a gene, *in-phase *(i.e., in phase 0) stop codons are generally not permitted in coding exons; for this reason, any potential stop codon (regardless of its signal score) will eclipse any preceding start codon or acceptor site (or, on the reverse strand, stop codon or donor site) in the corresponding phase. The algorithm shown in Figure 5 addresses this issue by dropping any fully eclipsed signal (i.e., eclipsed in all three phases) from its queue.

For the reverse strand, line 3 of eclipse() should be changed to:

ω ← (p-pos(s)-len(s) - 1) **mod**3;

where len(s) is the length of the consensus sequence for signal s (e.g., 3 for ATG). Note that by x**mod**3 we mean the *positive *remainder after division of x by 3; in some programming languages (such as C/C++), a negative remainder may be returned, in which case 3 should be added to the result.

A special case of eclipsing which is not handled by eclipse() is that which occurs when a stop codon straddles an intron; this can be handled fairly simply by checking for such when considering each donor signal as a prospective predecessor for an acceptor signal (or vice-versa on the reverse strand). As each predecessor is evaluated, the bases immediately before the donor and immediately following the acceptor are examined, and if a stop codon is formed, the predecessor is no longer considered eligible for selection in the corresponding phase.

As shown in Figure 5, when a signal has been eclipsed in all three phases it can be removed from its queue. In this way, as a signal falls further and further behind the current position in the sequence, the signal becomes more and more likely to be eclipsed in all three phases as randomly formed stop codons are encountered in the sequence, so that coding queues (e.g., those holding forward strand start codons and acceptors, or reverse strand donors and stop codons) tend not to grow without bound, but to be limited on average to some maximal load determined by the nucleotide composition statistics of the sequence. Because of this effect, the expected number of signals which must be considered during predecessor evaluation can be considered effectively constant in practice.

In the case of noncoding queues (e.g., those holding forward strand donors or stop codons, etc.), the assumption that noncoding features follow a geometric (i.e., exponentially decreasing) distribution allows us to limit these queues to a single element (per phase), because once a noncoding predecessor has been selected in a given phase, no other noncoding predecessor which has already been compared to the selected predecessor can ever become more attractive by virtue of its transition probability (since they are the same for signals of the same type, of which all the signals in a single queue are), its duration probability (since the geometric distribution ensures that their respective duration probabilities decrease at the same rate), nor its sequence probability (since any nucleotides encountered after seeing the two potential predecessors will affect their sequence scores identically).

Because the coding and noncoding queues are effectively limited to a constant load (as argued above), the expected processing time at each nucleotide is O(1) in practice and therefore the entire algorithm up to this point requires time O(*L*) for an input sequence of length *L *and a GHMM with a fixed number of states. It will be seen that the traceback procedure described below also requires time O(*L*), and so this is the time complexity of the PSA decoding algorithm for normal eukaryotic genomes (i.e., those not especially lacking in random stop codons).

**Figure 6:**
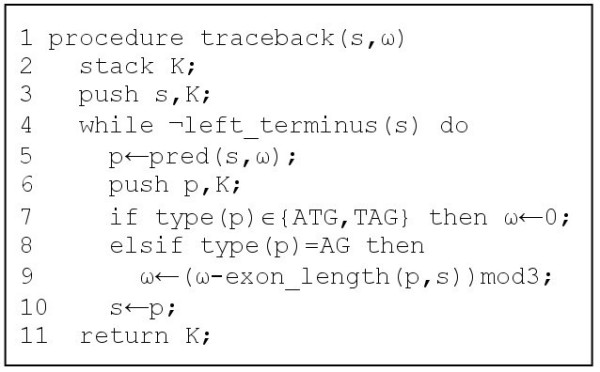
**The traceback() algorithm. **Reconstruction of the optimal parse by tracing back through trellis links. Parameters are the selected right-terminus signal s and its chosen phase ω. Returns a stack of signals constituting the optimal parse, with the top signal at the beginning of the parse and the bottom signal at the end. exon_length(p, s) denotes the number of coding nucleotides between p and s. The procedure operates by iteratively following the highest-scoring predecessor link from the current signal, adjusting the current phase as necessary when a trellis link corresponding to a coding feature is traversed.

Once the end of the sequence is reached, the optimal parse φ can be reconstructed by tracing back through the trellis links. In order for this to be done, a set of virtual, *anchor signals *(one of each type) must be instantiated at either terminus of the sequence (each having signal score *R*_*S *_= 0). Those at the left terminus will have been entered into the appropriate queues at the very start of the algorithm as prospective targets for the first trellis links (and having inductive scores *R*_*I *_= 0), and those at the right terminus are the last signals to be evaluated and linked into the trellis. The highest scoring of these right terminal anchor signals is selected (in its highest-scoring phase) as the starting point for the traceback procedure. Traceback consists merely of following the trellis links backward while adjusting for phase changes across exons, as shown in Figure 6.

Modifications to Figure 6 for features on the reverse-strand include changing the AG on line 8 to GT, changing the subtraction on line 9 to addition, and changing the 0 on line 7 to 2.

It should be clear from the foregoing that the space requirements of the PSA decoding algorithm are *O*(*L*|*Q*|) for sequence length *L *and variable-duration state set *Q*. If, for example, array elements are 8-byte double-precision floating point numbers, then the GHMM depicted in Figure 1 would require 14 prefix sum arrays (4 exon states × 3 phases + 1 intergenic state + 1 intron state), resulting in a memory requirement of at least 112 bytes per nucleotide. Generalizing this GHMM to handle both DNA strands would increase this to 216 bytes per nucleotide, so that processing of a 1 Mb sequence would require at least 216 Mb of RAM just for the arrays. Adding states for 5' and 3' untranslated regions would increase this to 248 Mb of RAM for a 1 Mb sequence, or over 1 Gb of RAM for a 5 Mb sequence. For the purposes of comparative gene finding on multiple organisms with large genes, these requirements seem less than ideal, especially when one considers the possibility of adding yet other states.

The memory requirements can be reduced in several ways. First, Markov chains can be shared by similar states. For example, the intron and intergenic states can share a single Markov chain trained on pooled noncoding DNA, and all the exon states can use the same three-periodic Markov chain trained on pooled coding DNA. To our knowledge, the extent to which this optimization affects the accuracy of the resulting gene finder has not been systematically investigated, though it is commonly used in practice. Second, the models for exons can be modified so as to utilize likelihood ratios instead of probabilities. If the models for exons are re-parameterized to compute:


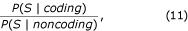


and the noncoding models are modified to compute:


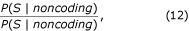


then the latter can be seen to be unnecessary, since it will always evaluate to 1. Such a modification is valid and will have no effect on the mathematical structure of the optimization problem given in Equation 2 as long as the denominator is evaluated using a Markov chain or other multiplicative model, since the effect of the denominator on inductive scores will then be constant across all possible predecessors for any given signal. Using such ratios allows us to skip the evaluation of all noncoding states, so that the number of prefix sum arrays required for a double-stranded version of the GHMM in Figure 1 would be only 6 (assuming the previous optimization is applied as well), corresponding to the three exon phases on two strands. Furthermore, to the extent that these likelihood ratios are expected to have a relatively limited numerical range, lower-precision floating point numbers can be used, or the ratios could instead be multiplied by an appropriate scaling factor and then stored as 2-byte integers [2]. This is a significant reduction, though asymptotically the complexity is still O(*L*|*Q*|). An additional consideration is that the log-likelihood strategy makes unavailable (or at least inseparable) the raw coding and noncoding scores, which might be desired later for some unforeseen application.

A third method of reducing the memory requirements is to eliminate the prefix sum arrays altogether, resulting in what we call the Dynamic Score Propagation (DSP) algorithm.

### The DSP decoding algorithm

Informally, the DSP algorithm is similar to the PSA algorithm except that rather than storing all nucleotide scores for all content sensors in a set of prefix sum arrays, we instead store only the specific elements of those arrays that are needed for assessing prospective predecessors during the trellis formation. Associated with each signal is a "propagator" variable which represents the log probability of the highest-scoring partial parse up to and including this signal. As processing proceeds left-to-right along the sequence, these propagators are updated so as to extend these partial parses up to the current position. In this way, the inductive score of each signal is incrementally propagated up to each potential successor signal that is encountered during processing; when a signal is eclipsed in all phases by stop codons (i.e., removed from its respective queue), propagation of that signal's inductive score halts, since further updates would be useless beyond that point. Because no prefix sum arrays are allocated, and because the signal queues are effectively limited in size (as argued previously), the expected memory requirements of DSP will be seen to be O(*L*+|*Q*|), where the constant factor associated with the *L *term is small, reflecting only the number of signals per nucleotide emitted by the signal sensors, as well as the memory required to store the sequence itself.

Let us introduce some notation. We define a *propagator *π to be a 3-element array, indexed using the notation π[*i*] for 0≤*i*≤2; when dealing with multiple propagators, π_*j*_[*i*] will denote element *i *of the *j*^th ^propagator.

Each signal *s*_*i *_will now have associated with it a propagator, denoted π_*i*_. For signals which can be members of multiple queues (such as start codons, which can be members of both the *initial exon *queue and the *single exon *queue), the signal will have one propagator per queue, but it will be clear from the context to which propagator we refer. Each queue will also have a propagator associated with it, though for the sake of reducing ambiguity we will refer to these as *accumulators *and represent them with the symbol α. The purpose of the accumulators is to reduce the number of updates to individual signal propagators; otherwise, every signal propagator in every queue would need to be updated at every position in the input sequence. The accumulator for a given queue will accumulate additions to be made to the propagators of the signals currently in the queue. The update of signal propagators from their queue's accumulator is delayed as long as possible, as described below. Accumulator scores are initialized to zero, as are the propagator scores for the left terminus anchor signals; the general case of propagator initialization will be described shortly.

Updating of a propagator π from an accumulator α is simple in the case of a noncoding queue:

∀_0≤ω≤2 _π[ω] ← π[ω] + α[0].     (13)

For coding queues, the update must take into account the location of the signal *s *associated with the propagator π, in order to synchronize the periodic association between phase and array index:

∀_0≤ω≤2 _π[ω] ← π[ω] + α[(ω - *pos*(*s*) - *len*(*s*))**mod**3],     (14)

or, on the reverse strand:

∀_0≤ω≤2 _π[ω] ← π[ω] + α[(ω + *pos*(*s*) + *len*(*s*))**mod**3].     (15)

Given a content sensor *M*, a coding accumulator can be updated according to the rule:

∀_0≤ω≤2 _α[ω] ← α[ω] + **log ***P*_*M*[(ω+*f*)**mod**3]_(*x*_*f*_),     (16)

or, on the reverse strand:

∀_0≤ω≤2 _α[ω] ← α[ω] + **log ***P*_*W*[(ω-*f*)**mod**3]_(*x*_*f*_),     (17)

where *f *is the position of the current nucleotide *x*_*f*_, *P*_*M*[ω]_(*x*_*f*_) is the probability assigned to *x*_*f *_by the content sensor *M *in phase ω, and *W *is the reverse-complementary model to *M *which computes the probability of its parameter on the opposite strand and taking contexts from the right rather than from the left. This update occurs once at each position along the input sequence. Use of *f *provides an absolute frame of reference when updating the accumulator. This is necessary because the accumulator for a queue has no intrinsic notion of phase: unlike an individual signal, a queue is not rooted at any particular location relative to the sequence.

For noncoding queues, only the 0^th ^element of the accumulator must be updated:

α[0] ← α[0] + **log ***P*_*M*_(*x*_*f*_).     (18)

All that remains is to specify the rule for selecting an optimal predecessor and using it to initialize a new signal's propagator. We first consider new signals which terminate a putative exon. Let *s*_*i *_denote the predecessor under consideration and *s*_*j *_the new signal. Denote by Δ the length of the putative exon. Then on the forward strand, we can compare predecessors with respect to phase ω via the scoring function *R*_*CI *_+ *R*_*D *_+ *R*_*T*_, where *R*_*D *_and *R*_*T *_are the duration and transition scores described earlier and *R*_*CI *_includes the content score and the inductive score from the previous signal:

∀_0≤ω≤2 _*R*_*CI*_(*s*_*i*_,ω) ← π_*i*_[(ω - Δ)**mod**3].     (19)

On the reverse strand we have:

∀_0≤ω≤2 _*R*_*CI*_(*s*_*i*_,ω) ← π_*i *_[(ω + Δ)**mod**3].     (20)

For introns it is still necessary to separate the three phase-specific scores to avoid greedy behavior, though the phase does not change across an intron, so no Δ term is necessary:

∀_0≤ω≤2 _*R*_*CI*_(*s*_*i*_,ω) ← π_*i*_[ω].     (21)

When the preceding feature is intergenic we need only refer to phase zero of the preceding stop codon:

*R*_*CI*_(*s*_*i*_,ω) ← π_*i*_[0],     (22)

or, on the reverse strand, phase 2 of the preceding start codon (since the leftmost base of the reverse-strand start codon will reside in phase 2).

Once an optimal predecessor with score *R*_*CI *_+ *R*_*D *_+ *R*_*T *_is selected with respect to a given phase ω, the appropriate element of the new signal's propagator can be initialized directly:

π_*j*_[ω] ← *R*_*CI*_(*s*_*i*_,ω) + *R*_*D*_(*s*_*i*_,*s*_*j*_) + *R*_*T*_(*s*_*i*_,*s*_*j*_) + *R*_*S*_(*s*_*j*_),     (23)

where *R*_*S*_(*s*_*j*_) = *P*(*context*(*s*_*j*_)|θ_*j*_) is the score assigned to the context window of the new signal *s*_*j *_by the appropriate signal sensor θ_*j*_. An exception to Equation 23 occurs when ω is not a valid phase for signal *s*_*j *_(e.g., phase 1 for a start codon), in which case we instead set π_*j*_[ω] to -∞.

One final complication arises from the fact that the algorithm, as we have presented it, does not permit adjacent signals in a prospective parse to have overlapping signal sensor windows; to allow such would be to permit double-counting of nucleotide probabilities, thereby biasing the probabilistic scoring function. It is a simple matter to reformulate the algorithm so that signal sensors score only the two or three consensus nucleotides of the signals under consideration; this would allow adjacent signals in a prospective parse to be as close as possible without actually overlapping (i.e., a single exon consisting of the sequence ATGTAG would be permitted, even if the start codon and stop codon context windows overlapped). However, doing so might be expected to decrease gene finder accuracy, for two reasons: (1) statistical biases occurring at fixed positions relative to signals of a given type can in general be better exploited by a signal sensor specifically trained on such positions than by a content sensor trained on data pooled from many positions at variable distances from the signal, and (2) in the case of Markov chains and Interpolated Markov Models, probability estimates for nucleotides immediately following a signal can be inadvertently conditioned on the few trailing nucleotides of the preceding feature (assuming the chain has a sufficiently high order), even though the models are typically not trained accordingly. For these reasons, we prefer to use signal sensors which impose a moderate margin around their respective signals, both to detect any biologically relevant biases which might exist within those margins, and to ensure that content sensors condition their probabilities only on nucleotides within the same feature.

Given the foregoing, it is necessary to utilize a separate "holding queue" for signals which have recently been detected by their signal sensors but which have context windows still overlapping the current position in the DSP algorithm. The reason for this is that propagator updates via Equations 13–15 must not be applied to signals having context windows overlapping any nucleotides already accounted for in the accumulator scores, since to do so would be to double-count probabilities. It is therefore necessary to observe the following discipline.

Associated with each signal queue *G*_*i *_there must be a separate *holding queue*, *H*_*i*_. When a signal is instantiated by a signal sensor it is added to the appropriate *H*_*i *_rather than to *G*_*i*_. As the algorithm advances along the sequence, at each new position we must examine the contents of each holding queue *H*_*i *_to identify any signal having a context window which has now passed completely to the left of the current position. If one or more such signals are identified, then we first update the propagators of all the signals in the main queue *G*_*i *_using Equations 13–15, then zero-out the values of the accumulator α_*i *_for that queue, and then allow the recently passed signals to graduate from *H*_*i *_to *G*_*i*_. Observe that at this point all the signals in *G*_*i *_have in their propagators scores which have effectively been propagated up to the same point in the sequence, and that point is immediately left of the current position; this invariant is necessary for the proper operation of the algorithm. All content sensors are then evaluated at the current position and their resulting single-nucleotide scores are used to update the accumulators for their respective queues. Finally, whenever it becomes necessary to evaluate the signals in some queue *G*_*i *_as possible predecessors of a new signal, we must first update the propagators of all the elements of *G*_*i *_as described above, so that the comparison will be based on fully propagated scores.

### Equivalence of DSP and PSA

We now give a proof that DSP is mathematically equivalent to PSA, since it may not be entirely obvious from the foregoing description. We will consider only the forward strand cases; the proof for the reverse strand cases can be derived by a series of trivial substitutions in the proof below.

To begin, we show by induction that the signal propagator π_*j*_[ω] for signal *s*_*j *_is initialized to the PSA inductive score *R*_*I*_(*s*_*j*_,ω). For the basis step, recall that the left terminus anchor signals were initialized to have zero scores in both PSA and DSP, regardless of whether a given signal began a coding or noncoding feature. In the case of coding features, substituting Equation 19 into Equation 23 yields:

π_*j*_[ω] ← π_*i*_[(ω - Δ)**mod**3] + *R*_*D*_(*s*_*i*_,*s*_*j*_) + *R*_*T*_(*s*_*i*_,*s*_*j*_) + *R*_*S*_(*s*_*j*_).     (24)

According to Equation 10, this initialization will result in π_*j*_[ω] = *R*_*I*_(*s*_*j*_,ω) only if:

π_*i*_[(ω - Δ)**mod**3] = *R*_*I*_(*s*_*i*_*,γ*_*i*_) + *R*_*C*_(*s*_*i*_,*s*_*j*_,ω),     (25)

where γ_*i *_= (ω - Δ)**mod**3 according to Equation 8. At the time that signal *s*_*j *_is instantiated by its signal sensor, π_*i *_has been propagated up to *e *= *wpos*(*s*_*j*_) - 1, the nucleotide just before the leftmost position of the context window for *s*_*j*_. By the inductive hypothesis, π_*i*_[γ_*i*_] was initialized to *R*_*I*_(*s*_*i*_,γ_*i*_). This initialization occurred at the time when the current DSP position was at the beginning of the predecessor's context window. Note, however, that π_*i *_effectively began receiving updates at position *b *= *wpos*(*s*_*i*_) + *wlen*(*s*_*i*_), the position immediately following the end of the signal's context window, at which point *s*_*i *_graduated from its holding queue. Thus, π_*i*_[γ_*i*_] will have accumulated content scores for positions *b *through *e*, inclusive. In order to establish Equation 25, we need to show that these accumulations sum to precisely *R*_*C*_(*s*_*i*_,*s*_*j*_,ω).

Substituting Equation 16 into Equation 14 we get the following formula describing propagator updates as if they came directly from content sensor *M*:

∀_0≤ω≤2 _π[ω] ← π[ω] + **log ***P*_*M*[(ω+*Δ*)**mod**3]_(*x*_*f*_),     (26)

where Δ = *f*-(*pos*(*s*_*i*_) + *len*(*s*_*i*_)) is the distance between the rightmost end of signal *s*_*i *_and the current position *f *in the DSP algorithm. Let us introduce the notation:

F(*i*,*j*,ω) = ∑_*k *= *i*..*j*_**log ***P*_*M*[(ω+*k*)**mod**3]_(*x*_*k*_).     (27)

Using this notation, π_*i*_[γ_*i*_] has since its initialization accumulated F(*b*,*e*,γ_*i *_- *pos*(*s*_*i*_) - *len*(*s*_*i*_)); this can be verified by expanding this expression via Equation 27 and observing that the result equals a summation of the log term in Equation 26 over *f *= *b *to *e*. Looking at init_phased(), it should be obvious that the effect of lines 5 and 8 will be that:

α_*i*,γ _[*h*] = ∑_*k *= 0..*h*_**log ***P*_*M *[(*k*+γ)*mod*3]_(*x*_*k*_) = F(0,h,γ).     (28)

According to Equation 5, showing that π_*i*_[γ_*i*_] has accumulated *R*_*C*_(*s*_*i*_,*s*_*j*_,ω) is therefore equivalent to:

*F*(*b*,*e*,ψ) = *F*(0,*wpos*(*s*_*j*_) - 1,γ) - *F*(0,*wpos*(*s*_*i*_) + *wlen*(*s*_*i*_) - 1,γ),     (29)

where ψ = γ_*i *_- *pos*(*s*_*i*_) - *len*(*s*_*i*_) and γ = ω - *pos*(*s*_*j*_). Equivalently:

*F*(*b*,*e*,ψ) = *F*(0,*e*,γ) - *F*(0,*b *- 1,γ).     (30)

To see that ψ ≡ γ(**mod**3), observe that *pos*(*s*_*j*_) - (*pos*(*s*_*i*_) + *len*(*s*_*i*_)) = Δ, the length of the putative exon (possibly shortened by three bases, in the case where *s*_*i *_is a start codon), and further that γ_*i *_- ω ≡ -Δ(**mod**3) according to Equation 8, so that ψ - γ ≡ Δ-Δ ≡ 0(**mod**3). Thus, Equation 30 is equivalent to:

*F*(*b*,*e*,γ) = *F*(0,*e*,γ) - *F*(0,*b *- 1,γ),     (31)

which can be established as a tautology by simple algebra after expansion with Equation 27. This shows that the signal propagator for signal *s*_*j *_is initialized to the PSA inductive score *R*_*I*_(*s*_*j*_,ω), and thus establishes the inductive step of the proof in the case of coding features.

To see that the above arguments also hold for noncoding features, note that Equation 21 simplifies Equation 25 to:

π_*i*_[ω] = *R*_*I*_(*s*_*i*_,ω) + *R*_*C*_(*s*_*i*_,*s*_*j*_),     (32)

that Equations 13 and 18 combine to simplify Equation 26 to:

∀_0≤ω≤2 _π[ω] ← π[ω] + **log ***P*_*M*_(*x*_*f*_),     (33)

and that lines 4 and 6 of init_nonphased() cause:

α_*i*_[*h*] = ∑_*k *= 0..*h*_**log ***P*_*M*_(*x*_*k*_) = F_*NC*_(0,*h*),     (34)

for *F*_*NC*_(*i*,*j*) = ∑_*k *= *i*..*j*_**log ***P*_*M*_(*x*_*k*_). We can thus reformulate Equation 29 as:

*F*_*NC*_(*b*,*e*) = *F*_*NC*_(0,*wpos*(*s*_cur_) - 1) - *F*_*NC*_(0,*wpos*(*s*_pred_) + *wlen*(*s*_pred_) - 1),     (35)

or, equivalently:

*F*_*NC*_(*b*,*e*) = *F*_*NC*_(0,*e*) - *F*_*NC*_(0,*b *- 1),     (36)

which is again a tautology. In the interests of brevity, we leave it up to the reader to verify that the above arguments still apply when the noncoding features are intergenic, thereby invoking Equation 22 rather than Equation 21 in formulating Equation 31.

To see that the selection of optimal predecessors is also performed identically in the two algorithms, note that the PSA criterion given in Equation 7 is equivalent to the argmax(*R*_*CI *_+ *R*_*D *_+ *R*_*T*_) criterion of DSP as long as *R*_*CI*_(*s*_*i*_,ω) = *R*_*C*_(*s*_*i*_,*s*_*j*_,ω) + *R*_*I*_(*s*_*i*_*,γ*_*i*_) at the time the optimal predecessor is selected, which we have in fact already shown by establishing Equation 25.

Thus, DSP and PSA build identical trellises; application of the same traceback() procedure should therefore produce identical gene predictions.

### Fast decoding of Markov chains

Markov chains are typically implemented in GHMM-based gene finders using hash tables, due to the simplicity of such an implementation. Thus, for a given Markov chain *M *we may utilize a hash table which associates the probability *P*_*M*_(*x*_*j*_|*x*_*j*-*n*_..*x*_*j*-1_) with the sequence *x*_*j*-*n*_..*x*_*j*_. Although hash tables provide a relatively efficient solution for this task, they are wasteful in the sense that as we evaluate the chain on successive nucleotides in a sequence, we repeatedly manipulate preceding nucleotides in forming successive substrings to be indexed into the hash table.

A much faster (and much more elegant) solution is to employ a Finite State Machine (FSM) in which states exist for all possible sequences of length *n*+1 or less, and where the state having label *x*_*j*-*n*_..*x*_*j *_emits the probability *P*_*M*_(*x*_*j*_|*x*_*j*-*n*_..*x*_*j*-1_), for *n*^th^-order Markov chain *M*. In this way, the transition probabilities of the Markov chain become the state emissions of the FSM. During a single left-to-right scan of a sequence, each base requires only a single two-dimensional array indexing operation to access the desired probability, and a single integer value store operation to remember the identity of the new state. When compared to the typical regime of arithmetic and bit-shift operations over an (*n*+1)-element string that would be required for a typical hash function, the difference can be significant.

Implementing this optimization is fairly straightforward, both for conventional Markov chains and for Interpolated Markov Models, whether homogeneous or three-periodic. Central to the method is a means of mapping between state labels and integer state identifiers for use in indexing into the transition table. The base-4 number system can be utilized for this purpose, assuming a nucleotide mapping such as ∇ = {A↔0, C↔1, G↔2, T↔3}. To account for lower-order states, define:


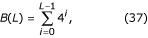


which gives the total number of strings of length less than *L*. Converting a string *S *= *x*_0_..*x*_*L*-1 _to base-4 can be accomplished as follows:


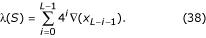


Now a string *S *can be mapped to a state index using:

*state*(*S*) = *B*(|*S*|) + λ(*S*),     (39)

where |*S*| denotes the length of *S*.

Given this integer↔label mapping and an *n*^th^-order Markov chain in hash table format, the FSM state emissions can be initialized by indexing state labels into the hash table to obtain the Markov chain transition probabilities. The transition table can be initialized fairly simply by noting that the successor of state *x*_0_..*x*_*L*-1 _upon seeing symbol *s *is *x*_1_..*x*_*L*-1_*s *if *L *= *n *+ 1, or *x*_0_..*x*_*L*-1_*s *for *L *<*n *+ 1. A model for the reverse strand can be handled by applying this scheme in reverse, so that the state with label *x*_*j*-*n*_..*x*_*j *_emits the probability *P*_*M*_(*x*_*j*-*n*_|*x*_*j*-*n*+1_..*x*_*j*_), and the lower-order states are reserved for the end of the sequence rather than the beginning.

## Results

Table 1 shows the memory and time requirements for two GHMM gene finders, one using the PSA algorithm and the other the DSP algorithm, on a 922 Kb sequence. Note that the DSP gene finder has 31 states, while the PSA gene finder explicitly evaluates only 6 states, so that they both give a ratio of 2.8 seconds per state on this sequence, while the ratio of memory per state is 14 Mb for the PSA gene finder and 0.95 Mb for the DSP gene finder. Thus, the DSP and PSA algorithms appear to consume the same amount of time per state, while DSP requires only a fraction of the memory (per state) as PSA.

Table 2 shows the results of applying the FSM optimization to a DSP gene finder to accelerate its content sensors. As can be seen from the table, the FSM approach reduces execution time by more than half (as compared to a hash table implementation), while also reducing total RAM usage. The DSP/FSM configuration reported here utilized both conventional Markov chains as well as Interpolated Markov Models, both represented using FSMs. Note that the hashing software used for comparison was a very efficient implementation which used native C character arrays; in particular, we did *not *use the C++ Standard Template Library (STL) implementations of *string *and *hash*, due to efficiency concerns regarding the re-copying of string arguments to the hash function. Our custom string hashing implementation was found to be much faster than the STL implementation (data not shown). Accordingly, one can expect an FSM implementation to show even greater gains as compared to an STL-based hashing implementation.

We utilized our DSP-based gene finder TIGRscan [5] in the construction of our syntenic gene finder TWAIN, a Generalized Pair HMM which performs gene prediction in two genomes simultaneously. TWAIN operates by invoking a modified version of TIGRscan to build a directed acyclic graph of all high-scoring parses of each of the two input sequences. Early experiments indicated that these parse graphs could be quite large in practice and may therefore require a significant portion of available RAM for their storage. In addition, the dynamic programming matrix used by TWAIN promised to be large as well. It was in anticipation of this problem that we were prompted to develop TIGRscan using the DSP architecture, to minimize the memory requirements of the underlying GHMM, thereby freeing the remaining available memory for use by the rest of the machinery within TWAIN.

As a result of these and other optimizations (such as our use of a sparse matrix representation for TWAIN's dynamic programming algorithm) we were able to apply TWAIN's gene prediction component to a pair of fungal genomes (*Aspergillus fumigatus *and *A. nidulans*) while consuming under 50 Mb of RAM, whereas an earlier prototype of this system applied to the same input data routinely exhausted all available memory on a computer with 1 Gb of RAM. We are hopeful that through the use of optimizations such as those described here we will be able to apply TWAIN to other pairs of genomes with longer genes, and possibly extend the program to handle more than two species simultaneously.

## Conclusions

In describing a number of optimizations for GHMM-based gene finders and making available two complete open-source software systems embodying these methods, it is our hope that others will be more enabled to explore promising extensions to the GHMM framework, thereby improving the state-of-the-art in gene prediction techniques.

## Availability and requirements

* **Project name: **TIGRscan, GlimmerHMM

* **Project home page: **

* **Operating system(s): **Linux/UNIX

* **Programming language: **C/C++

* **Other requirements: **compiled using gcc 3.3.3

* **License: **Artistic License, see 

* **Any restrictions to use by non-academics: **terms of Artistic License

## Authors' contributions

The DSP algorithm was devised by WHM, who also performed the computational experiments and wrote the manuscript. The PSA gene finder GlimmerHMM was implemented by MP. MP, ALD, and SLS provided detailed insights into the PSA architecture and provided valuable comments on the manuscript.

## Figures and Tables

**Table 1:** Space and time requirements for two gene finders Two gene finders, the 31-state DSP gene finder TIGRscan, and the 6-state PSA gene finder GlimmerHMM, were run on a 922 Kb sequence. The DSP gene finder used raw probabilities and the PSA gene finder used log-likelihood ratios. The DSP implementation required less memory, both in total and per state, than the PSA implementation. Although the PSA implementation required less total time, the DSP implementation required the same amount of time per state, so that for a given gene finder with a fixed number of states, DSP decoding can be expected to be fully as fast as PSA decoding.

	**RAM (Mb)**	**RAM/state (Mb)**	**Time, min:sec**	**seconds/state**
**31-state DSP**	29	0.95	1:28	2.8
**6-state PSA**	84	14	0:17	2.8

**Table 2:** Efficiency of Markov chain implementations Execution time for a 31-state GHMM gene finder utilizing hash tables or FSMs for its content sensors, applied to a 1.8 Mb sequence. The FSM implementation was over twice as fast as the hash table implementation, and required significantly less memory.

	**time (min:sec)**	**total RAM**
**DSP/Hash**	1:15	53 Mb
**DSP/FSM**	0:34	44 Mb

## References

[B1] Kulp D, Haussler D, Reese MG, Eeckman FH (1996). A generalized hidden Markov model for the recognition of human genes in DNA. Proc Int Con Intell Syst Mol Biol.

[B2] Burge C (1997). Identification of Genes in Human Genomic DNA. PhD Thesis.

[B3] Cawley SE, Wirth AI, Speed TP (2001). Phat – a gene finding program for *Plasmodium falciparum*. Mol Biochem Parasitol.

[B4] Stanke M, Waack S (2003). Gene prediction with a hidden Markov model and a new intron submodel. Bioinformatics.

[B5] Majoros WM, Pertea M, Salzberg SL (2004). TIGRscan and GlimmerHMM: two open-source *ab initio *eukaryotic gene finders. Bioinformatics.

[B6] Korf I (2004). Gene finding in novel genomes. BMC Bioinf.

[B7] Pachter L, Alexandersson M, Cawley S (2002). Applications of generalized pair hidden Markov models to alignment and gene finding problems. J Comput Biol.

[B8] Alexandersson M, Cawley S, Pachter L (2003). SLAM: Cross-species gene finding and alignment with a generalized pair hidden Markov model. Genome Res.

[B9] Rabiner LR, Juang B-H (1986). An introduction to hidden Markov models. IEEE Transactions on Acoustics Speech, Signal Processing.

[B10] Rabiner LR (1989). A tutorial on hidden Markov models and selected applications in speech recognition. Proceedings of the IEEE.

[B11] Salzberg SL, Searls DB, Kasif S (1998). Computational Methods in Molecular Biology.

[B12] Durbin R, Eddy S, Krogh A, Mitchison G (1998). Biological Sequence Analysis.

[B13] Burge C, Karlin S (1997). Prediction of complete gene structures in human genomic DNA. J Mol Biol.

[B14] Salzberg SL, Pertea M, Delcher AL, Gardner MJ, Tettelin H (1999). Interpolated Markov models for eukaryotic gene finding. Genomics.

[B15] Majoros WM, Pertea M, Salzberg SL (2005). Efficient implementation of a centralized Pair. Hidden Markov Model for comparative gene finding. Bioinformatics.

